# A retrospective controlled clinical study of Cobb angle distribution of the main thoracic curve in adolescent idiopathic scoliosis

**DOI:** 10.1097/MD.0000000000011473

**Published:** 2018-07-13

**Authors:** Jian Zhao, Jianping Fan, Yuanyuan Chen, Changwei Yang, Gengwu Li, Ming Li

**Affiliations:** a Department of Orthopedics, Changhai Hospital of the Second Military Medical University, Shanghai; b Department of Orthopedics of Jinling Hospital, Nanjing University, School of Medicine, Nanjing, Jiangsu; c Department of Orthopedics, Handan No. 285 Hospital of the Chinese People's Liberation Army, Handan, Hebei; d Department of Laboratory Medicine, Changhai Hospital, Shanghai; e Panzhihua Central Hospital, Panzhihua, China.

**Keywords:** adolescent idiopathic scoliosis, correction rate, flexibility, pedicle screw

## Abstract

To compare the characteristics of Cobb angle distribution of the main thoracic curve (MTC) in patients with Lenke 1 adolescent idiopathic scoliosis (AIS) and differences in fulcrum-bending flexibility, correction rate, and correction index between different segments.

Included in this study were 40 consecutive patients with Lenke 1 AIS who received posterior correction and fusion with pedicle screws. Cobb angle based on the proximal (T5–T7 or T6–T8), apical (T7–T9 or T8–T10), and distal (T9–T11 or T10–T12) segments in the fulcrum-bending position was measured before and after surgery. The flexibility ([Cobb angle of each segment − residual Cobb angle on fulcrum bending]/Cobb angle of each segment × 100%), correction rate ([Cobb angle of each segment − postoperative residual Cobb angle]/Cobb angle of each segment × 100%]), and correction index (correction rate of each segment/preoperative flexibility of each segment) in different segments were calculated. Comparative analyses were conducted by variance analysis.

The mean age before surgery, Cobb angle, Risser sign, and follow-up time were 14.15 ± 2.13 years, 51.17 ± 10.72°, 2.78 ± 1.73, and 43.75 ± 9.82 months, respectively. MTC Cobb angle of the proximal segments was similar to that of the distal ones (12.88 ± 4.81 vs 12.85 ± 5.00) versus 25.45 ± 5.90 in the middle segments (*P* < .001). The flexibility was higher in the distal segments than that in the proximal or apical segments (66.43 ± 0.22% vs 43.78 ± 0.20% or 32.55 ± 0.17%, *P* < .001). One week after surgery, the correction rate in these 3 segments was 69.55 ± 0.1%, 66.25 ± 0.17%, and 75.28 ± 0.16 (*P* = .067), and the correction index was 2.15 ± 1.78, 3.16 ± 3.60, and 1.53 ± 1.93 (*P* = .019); the correction rate during the 3-year follow-up period was 68.06 ± 0.19%, 69.98 ± 0.15%, and 73.29 ± 0.17 (*P* = .212); and the correction index was 2.12 ± 1.78, 3.20 ± 3.54, and 1.49 ± 1.93 (*P* = .012), respectively.

The proximal, apical, and distal segments in Lenke 1 AIS accounted for about 25%, 50%, and 25% of MTC Cobb angle, respectively. The distal segments were found to be most flexible and the apical segments most rigid. The correction rate was similar between the proximal, apical, and distal segments, and the correction index in the apical segments was higher than that in the proximal and distal segments.

## 1 Introduction

Correction surgery is often required to reconstruct the spinal alignment in patients with severe deformities due to adolescent idiopathic scoliosis (AIS).^[1]^ Before surgery, severity of the spinal deformity needs to be accessed via Cobb angle-based posteroanterior chest radiography.^[2]^ In addition, flexibility should be estimated to plan the surgical approach, knowing that it is essential for selection of the lower instrumented vertebra and the upper instrumented vertebra.^[3]^ To some extent, flexibility assessment can also predict the surgical outcome.

There are several methods to assess curve flexibility, including supine lateral bending, fulcrum bending, manual correction, and traction methods.^[4]^ Cobb angle on supine lateral bending is a common index to assess the flexibility of scoliosis in the coronal plane, and the postoperative residual Cobb angle is an imaging index to predict the surgical outcome, especially in patients with Lenke 5 AIS.^[5]^ With the improvement of all-pedicle screw instrumentation, prediction based on the lateral-bending film may fail to reflect the surgical outcome.^[4]^ For those proximal main thoracic curves (MTCs), the fulcrum bending film can reveal flexibility more accurately as compared with the lateral-bending film,^[6]^ particularly for curves between 40° and 65°.^[7]^

Although there have been many studies on flexibility assessment of the scoliotic spine and the surgical correction rate of diverse instrumentations, few studies have investigated the distribution characteristics of Cobb angle in the MTC. Hasler et al^[8]^ first broke the term “structurality” down to a segmental level in Lenke 1 AIS patients. The segmental correction rate based on fulcrum-bending films indicated the similar structurality tethering within the 4 periapical segments. However, there is a lack of information about the characteristics of MTC. What is the proportion of the proximal, apical, and distal segments against the overall MTC Cobb angle? Is there any significant difference in flexibility between these different segments in the fulcrum-bending position? Is there any significant difference in the correction rate between different segments? To answer these questions, we conducted a retrospective clinical study to analyze the characteristics of MTC Cobb angle distribution in Lenke 1 AIS and compare differences in flexibility and the correction rate between the proximal, middle, and distal segments.

## 2 Materials and methods

### 2.1 Patient population

The study was approved by the Ethics Institutional Review Board of Chang Hai Hospital. This study included 40 Lenke 1 AIS patients. The inclusion criteria were patients with Lenke 1 AIS with MTC Cobb angle involved in T5–T11 or T6–T12; patients receiving single-stage posterior correction surgery with pedicle screws; patients with no previous medical history of spinal surgery and manipulations that may affect the flexibility; patients receiving no spinal osteotomy; and patients with availability of sufficient X-ray films comprising standing posteroanterior, lateral, and fulcrum-bending X-ray films before surgery, and standing posteroanterior and lateral X-ray films 5 to 7 days after surgery and at the final follow-up visit.

### 2.2 Radiographic measurement

**Figure 1:**
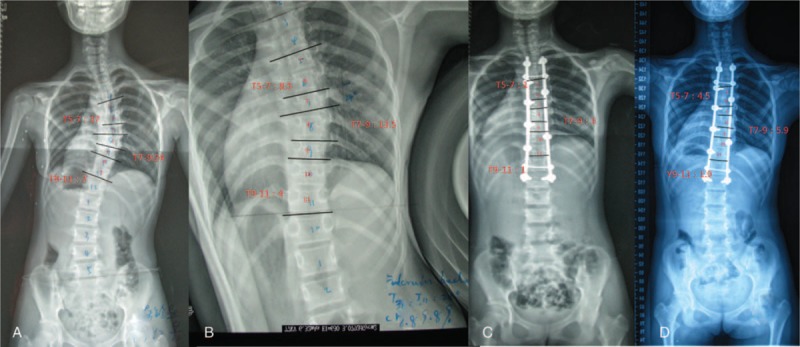
Cobb angle of the proximal (T5–T7), apical (T7–T9), and distal (T9–T11) segments of the main thoracic curve (T5–T12) is measured. A, B, C and D indicate the posteroanterior X-ray film before surgery, fulcrum-bending film and posteroanterior film 5 days after surgery, and posteroanterior film at the end of the 3-year follow-up period, respectively.

MTC Cobb angles in the proximal (T5–T7 or T6–T8, according to the end vertebra), apical (T7–T9 or T8–T10), and distal (T9–T11 or T10–T12) segments were measured on the fulcrum-bending and posterior–anterior films before and immediately after surgery and at the final follow-up visit (Fig. 1). Each parameter was measured twice by 2 radiologists independently, and the mean value of the 4 measurements was accepted for further analysis. Any discrepancy was omitted through discussion.

The fulcrum-bending films were used to estimate preoperative flexibility in these different segments.^[9,10]^ The postoperative correction rate and the correction index were also calculated as follows.^[11]^

Preoperative flexibility (%) = (Cobb angle of each segment − residual Cobb angle on fulcrum-bending bending)/Cobb angle of each segments × 100%.

Postoperative correction rate = (Cobb angle of each segment − residual postoperative Cobb angle)/Cobb angle of each segment × 100%.

Correction index = Correction rate of each segment/Preoperative flexibility of each segment.

### 2.3 Surgical procedures

All the surgical procedures were performed by the same surgeon from the same institution. All patients received posterior correction and fusion surgery with pedicle screws under controlled hypotension anesthesia. In most patients, the screws were placed on at least the entire concave side, and translation combined with the rotating rod-derotation technique was used to correct the spinal deformity. Freeze-dried allogeneic bone was used as the fusion material.

### 2.4 Statistical analysis

Statistical software SPSS 17.0 was used to perform comparative analysis. Variance analysis coupled with least significant difference-T test was used to compare differences in Cobb angle distribution, fulcrum-bending flexibility, correction rate, and correction index immediately after surgery and at the final follow-up visit between the different segments. A 2-sided *P* value < .05 was considered statistically significant.

## 3 Results

The mean age of the 40 included patients was 14.15 ± 2.13 years at the time of surgery, including 33 (82.5%) women and 7 (17.5%) men. The mean Risser sign was 2.78 ± 1.73. The mean follow-up time was 43.75 ± 9.82 months. The characteristics of the included patients are shown in Table 1.

**Table 1:**
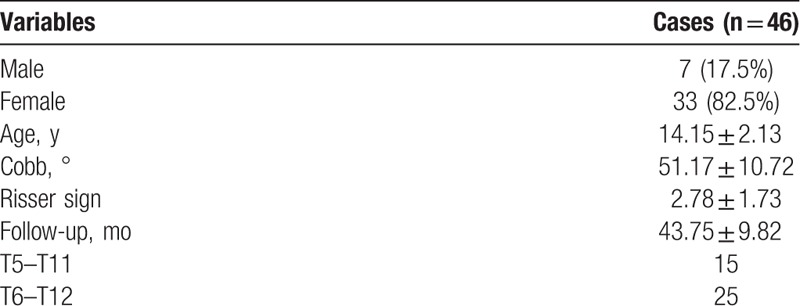
Demographics and clinical characteristics.

**Figure 2:**
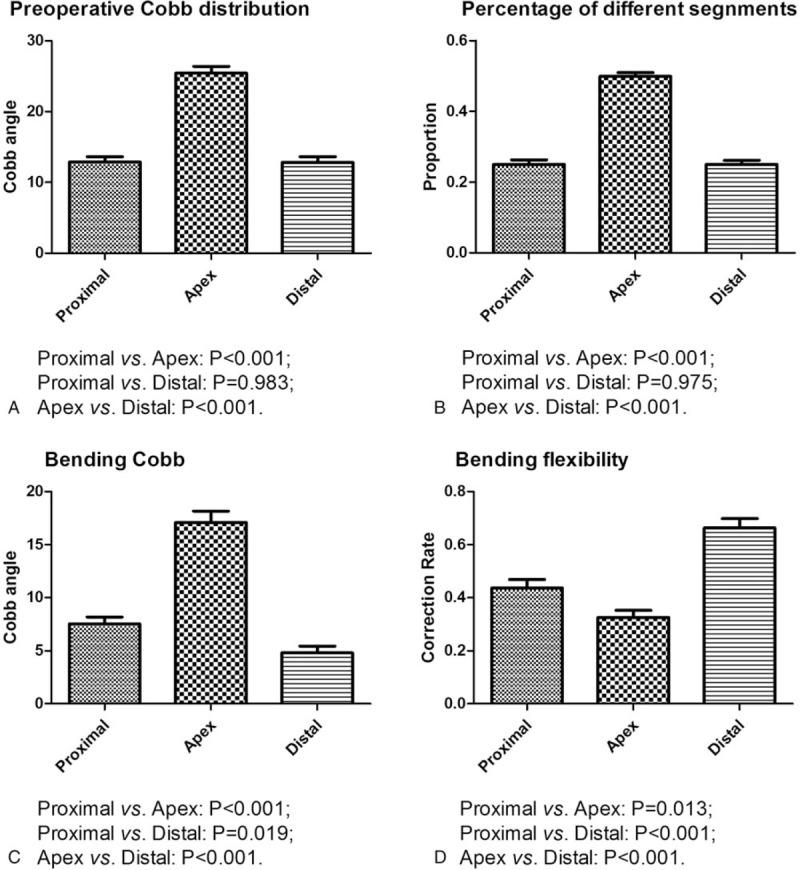
(A) Preoperative Cobb angle in the proximal (T5–T7 or T6–T8), apical (T7–T9 or T8–T10), and distal (T9–T11 or T10–T12) segments of main thoracic curve. (B) Percentage distribution of preoperative Cobb angle of the proximal, apical, and distal segments. (C) Fulcrum-bending Cobb angle of the proximal, apical, and distal segments. (D) Flexibility of the proximal, apical, and distal segments.

This study detected statistical significance in Cobb angle distribution between proximal (12.88 ± 4.81), apical (25.45 ± 5.90), and distal segments (12.85 ± 5.00) (*P* < .001). Multiple comparisons revealed a higher Cobb angle in the apical segments (proximal vs apical, *P* < .001; apical vs distal, *P* < .001), showing no significant difference between the proximal and distal segments (*P* = .983) (Fig. 2A). The similar finding was observed in terms of the percentage between the proximal (25.12 ± 0.78%), apical (49.95 ± 0.68%), and distal segments (25.08 ± 0.07%) (*P* < .001) (Fig. 2B).

A significant difference in bending Cobb was also observed between the proximal (7.53 ± 4.10), apical (17.10 ± 6.67), and distal segments (4.80 ± 4.10) (*P* < .001). Multiple comparisons revealed a higher bending Cobb angle in the apical segments (proximal vs apical, *P* < .001; apical vs distal, *P* < .001), and a relatively higher bending Cobb angle in the proximal segments as compared with that in the distal segments (*P* = .019) (Fig. 2C). A similar difference in flexibility was also observed between the proximal (43.78 ± 0.20%), apical (32.55 ± 0.17%), and distal segments (66.43 ± 0.22%) (*P* < .001) (Fig. 2D).

**Figure 3:**
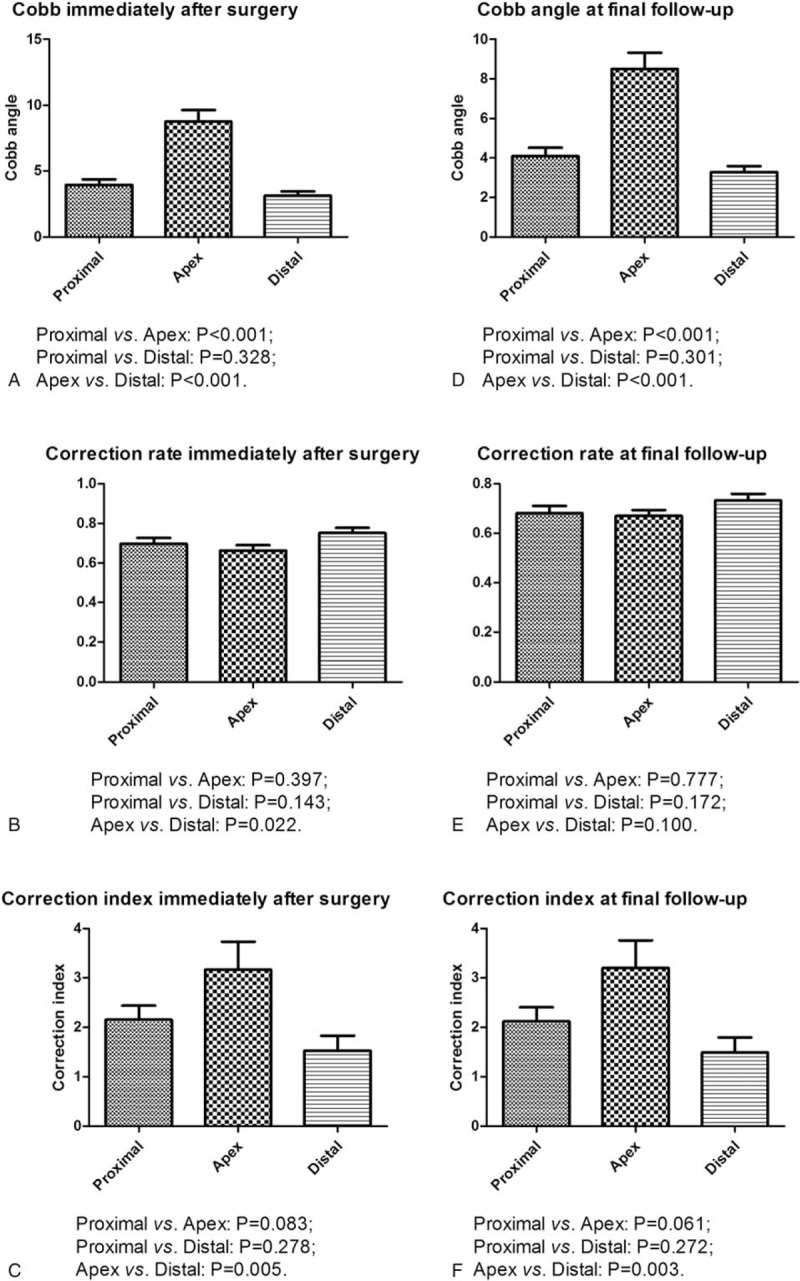
(A) Cobb angle in the proximal (T5–T7 or T6–T8), apical (T7–T9 or T8–T10), and distal (T9–T11 or T10–T12) segments of MTC 1 week after surgery. (B) Correction rate of the proximal, apical, and distal segments 1 week after surgery. (C) Correction index of the proximal, apical, and distal segments 1 week after surgery. (D) Cobb angle in the proximal, apical, and distal segments of MTC at the final follow-up visit. (E) Correction rate of the proximal, apical, and distal segments at the final follow-up visit. (F) Correction index of the proximal, apical, and distal segments at the final follow-up visit. MTC = main thoracic curve.

A difference in Cobb angle was observed immediately after surgery between the proximal (3.95 ± 2.64), apical (8.75 ± 5.58), and distal segments (3.13 ± 2.04) (*P* < .001). Multiple comparisons revealed a higher Cobb angle in the apical segments (proximal vs apex, *P* < .001; apical vs distal, *P* < .001), while there was no significant difference between the proximal and distal segments (*P* = .328) (Fig. 3A). In addition, there was no significant difference in the correction rate between the proximal (69.55 ± 0.19%), apical (66.25 ± 0.17%), and distal segments (75.28 ± 0.16) (*P* = .067) (Fig. 3B). A relatively higher correction index was observed in the apical segments as compared with that in the distal segments (3.16 ± 3.60 vs 1.53 ± 1.93, *P* = .005) (Fig. 3C).

There were differences in Cobb angle at the final follow-up visit between the proximal, apical, and distal segments (4.10 ± 2.63, 8.50 ± 5.51, and 3.27 ± 1.94, *P* < .001). Multiple comparisons revealed a higher bending Cobb angle in the apex segments (proximal vs apical, *P* < .001; apical vs distal, *P* < .001), while there was no difference between the proximal and distal segments (*P* = .301) (Fig. 2D). In addition, there was no significant difference in the correction rate between the proximal (68.06 ± 0.19%), apical (69.98 ± 0.15%), and distal segments (73.29 ± 0.17) (*P* = .212) (Fig. 2E). The correction index was 2.12 ± 1.78, 3.20 ± 3.54, and 1.49 ± 1.93 for the proximal, apical, and distal segments, respectively (*P* = .012). Multiple comparisons revealed a relatively higher correction index in the apex segments as compared with that in the distal segments (3.20 ± 3.54 vs 1.49 ± 1.93, *P* = .003) (Fig. 3F).

## 4 Discussion

AIS is a 3D deformity of the spine with unknown etiology. There has been ample research on the curve location, structural features, and natural history of scoliosis.^[12,13]^ A study on MTC in Lenke 1 AIS^[8]^ reported that the significant homogenous segmental tethering was confined to 4 periapical levels. The advent of modern surgical technologies and potent correction instrumentations has provided various surgical options for the correction of scoliosis. However, these methods for assessment of flexibility may fail to predict the correction outcome of all-pedicle screw instrumentation.^[4]^ In the present study, we found that MTC Cobb angle distribution seemed to be in a naturally symmetric state, meaning that the proportion of the proximal segment is similar to the distal part, accounting for about 25% of Cobb angle (Fig. 2B). The apical segment (the more rigid and structurally scoliotic portion) accounted for about 50% (Fig. 2B). Flexibility distribution in the fulcrum-bending position showed that the distal segments were more flexible than the proximal ones, and the apical segments had the poorest flexibility (Fig. 2D), which might result from the discrepant mobility of different intervertebral disks. In general, the lower the vertebral disks are located, the higher movability they would have due to their larger size as compared with the proximal segments. In addition, structural changes mainly occurred in the apical segments, which accounts for the poorest flexibility there.^[8]^

It was previously reported^[14]^ that a higher flexibility often indicated a higher correction rate. However, we found no significant difference in the correction rate between the proximal, apical, and distal segments, suggesting that the currently available methods for assessment of curve flexibility may not be able to predict the surgical outcome of all-pedicle screw instrumentation.^[4]^ In addition, Vora et al^[11]^ proposed to assess the correction by a ratio (correction rate of each segment/preoperative flexibility of each segment). Using this correction index, we can truly evaluate and compare the correction ability between different constructs. We found that the correction index was the highest in the apical segment, probably due to the strong correction power of the modern instrumentations. In addition, surgeons frequently tend to use multiple techniques such as local release, distraction, and compression techniques to implement correction in the apical segment. Even though no spine osteotomy was performed in this study, we could infer that except for the apical region, spine osteotomy may not be necessary for all other segments.

Several recent studies^[15–17]^ have suggested that there is no need to implant pedicle screws in every vertebra when posterior correction surgery is performed for the treatment of mild-to-moderate AIS. Fewer screws could be used without affecting the correction outcome.^[17]^ Nevertheless, there is currently no reference guideline for selecting the location of screw implantation. The current study may provide some references for reducing the use of pedicle screws. We also found that the apical vertebral segments made the greatest contribution to the correction index of MTC (Fig. 3C and F), and theoretically it would bear more stress.^[18]^ In our opinion, screws used in the apical region should have higher strength, and the number of screws implanted in the relatively flexible distal segments could be reduced.

Despite our successful qualitative assessment on MTC Cobb angle distribution, several limitations should be taken into consideration. First, the statistical power might be dwarfed for the relatively small number of patients included in this study. Second, some patients failed to provide data about the health-related quality of life due to varying reasons. Third, some patients included in this study had relatively satisfactory flexibility, and therefore some of our conclusions may not be appropriate to patients with more severe rigidity. Fourth, we rarely required AIS patients to undergo radiography in hyperextension and hyper-flexion positions in our institution, and therefore the present study did not compare differences in sagittal flexibility between different segments. Finally, this is a single-center study.

In summary, the proximal, apical, and distal segments in Lenke 1 AIS accounted for about 25%, 50%, and 25% of MTC Cobb angle, respectively. Differences in fulcrum-bending flexibility demonstrated that the distal segments were more flexible than the proximal ones, and the apical segments had the poorest flexibility. There was no significant difference in the correction rate between the proximal, apical, and distal segments, and the apical segments had a relatively higher correction index.

## Author contributions

**Conceptualization:** Gengwu Li, Ming Li.

**Data curation:** Yuanyuan Chen, Changwei Yang, Gengwu Li.

**Funding acquisition:** Ming Li.

**Investigation:** Jianping Fan.

**Methodology:** Changwei Yang, Gengwu Li.

**Project administration:** Ming Li.

**Resources:** Changwei Yang, Ming Li.

**Software:** Jian Zhao.

**Supervision:** Gengwu Li, Ming Li.

**Validation:** Jianping Fan.

**Writing – original draft:** Jian Zhao.

**Writing – review and editing:** Jianping Fan, Yuanyuan Chen.
